# Reproductive Challenges in Mutated BRCA Gene Carriers: A Narrative Review

**DOI:** 10.3390/cimb48080843

**Published:** 2026-08-19

**Authors:** Maria Vouza, Maria Papadoliopoulou, Konstantinos Dimitrakakis, Georgios Zografos, Nikolaos V. Michalopoulos, Nikolaos Arkadopoulos

**Affiliations:** 1 6th Department of Obstetrics & Gynaecology, General and Maternity Hospital Elena Venizelou, 11521 Athens, Greece; 2 4th Department of Surgery, Attikon University Hospital, 12462 Athens, Greece; 3 1st Department of Obstetrics and Gynaecology, Alexandra Hospital, 11528 Athens, Greece; 4 1st Propaedeutic Department of Surgery, Hippocration General Hospital, 11527 Athens, Greece

**Keywords:** *BRCA* genes, mutation carrier, fertility, breast cancer, assisted reproduction

## Abstract

The Breast Cancer (*BRCA*) genes play an important role in repairing double-strand deoxyribonucleic acid (DNA) breaks. Mutations in these genes are associated with the development of malignancies, most notably breast and ovarian cancer. Carriers of these mutations are often faced with issues that have serious implications for their reproductive choices, including the occurrence and treatment of breast or ovarian cancer, recommendations for preventive mastectomy and salpingo-oophorectomy, and the risk of transmitting the *BRCA* gene mutation to an offspring. These issues often lead to the search for methods of fertility preservation or assisted reproduction. This review aims to highlight and explain all the ways in which *BRCA* gene mutations affect fertility in women. Specifically, the effect of *BRCA* carriage is assessed at the molecular, histological, clinical, and laboratory levels. Next, fertility preservation methods in carriers are analyzed and evaluated in terms of their safety and effectiveness. In addition, the effect of systemic therapies for breast cancer on ovarian reserves is explained. Finally, the possibility of preimplantation diagnosis of *BRCA* gene carriage is also examined.

## 1. Introduction

The *BRCA1* and *BRCA2* genes are among the most important genes associated with hereditary breast and ovarian cancer. They were first described in 1994 and 1995, respectively, following the observation that breast cancer occurs with greater frequency in certain families. These genes are located on chromosomes 17 and 13, respectively. They are tumor suppressor genes that produce proteins playing a crucial role in repairing DNA mutations in cells [1]. These proteins play a central role in maintaining genomic stability by repairing DNA double-strand breaks through the homologous recombination pathway. Following DNA damage, *BRCA* proteins participate in DNA damage response, activation of cell-cycle checkpoints, and recruitment of DNA repair proteins, including RAD51, thereby ensuring accurate DNA repair [2]. When DNA damage cannot be adequately repaired, apoptotic pathways are activated to eliminate genetically unstable cells [3]. Consequently, pathogenic variants in *BRCA1* or *BRCA2* impair homologous recombination, leading to genomic instability and increased cancer susceptibility. Given the fundamental role of *BRCA* proteins in preserving genomic integrity, their dysfunction has also been implicated in reproductive biology, providing the biological basis for the reproductive challenges discussed in the following sections [1]. The percentage of people who carry these specific mutations is 0.25%, or 1 in 400 individuals, while in certain ethnic groups these mutations are found more frequently, such as Ashkenazi Jews, where this percentage rises to 2%. A multitude of different mutations affecting these specific genes have been identified, and it has also been found that certain populations carry mutations that are not commonly found in other populations [4]. Cases of hereditary breast cancer account for only 5–10% of all breast cancer cases, suggesting that breast cancer is mostly sporadic. However, individuals who carry one of these two genes are at particularly high risk of developing breast and ovarian cancer. Specifically, *BRCA1* carriers have a ~72% chance of developing breast cancer and a ~44% chance of developing ovarian cancer. For *BRCA2* carriers, the incidence rates for these specific cancers are approximately ~69% and ~17%, respectively [5]. The risk of developing breast cancer in the general population is 12%, while for ovarian cancer the rate is 1% [6]. *BRCA* carriers tend to develop the disease at a younger age compared with patients without mutations, while carriers are also at particularly high risk of developing a new malignancy in the other breast, specifically 25–40% over a 20-year period [4]. In this population, especially in *BRCA1* carriers, the most common histological type is triple-negative cancer, which tends to be more aggressive [7]. *BRCA* genes have also been implicated in other malignancies, most notably primary peritoneal cancer, prostate cancer, and pancreatic cancer. There also appears to be an association between *BRCA* genes and the occurrence of esophageal, stomach, and biliary tract cancers [8].

There are three approaches to managing *BRCA* mutation carriers. The first is conservative management, which involves more intensive and earlier monitoring, specifically annual breast magnetic resonance imaging (MRI) at age 25, and after age 30, a combination of annual breast MRI and digital mammography until age 75. Clinical examination is recommended to be performed in addition to the above imaging methods every 6–12 months starting at age 25 [9]. As for ovarian cancer, no equally effective screening method has been identified. Some organizations recommend regular screening of the internal reproductive organs using transvaginal ultrasound and regular measurements of the cancer antigen 125 (CA-125) serum marker [10]. The second approach is surgical. Specifically, it involves bilateral mastectomy with or without reconstruction. With regard to ovarian cancer, bilateral salpingo-oophorectomy is the most effective and appropriate method of ovarian cancer prevention. The recommended age group for this procedure is 35–40 for *BRCA1* carriers and 40–45 for *BRCA2* carriers [9]. The third approach involves chemoprevention. The drugs approved by the Food and Drug Administration (FDA) for reducing the risk of breast cancer are the Selective Estrogen Receptor Modulators (SERMs) tamoxifen and raloxifene and are more beneficial for *BRCA2* carriers, due to the higher frequency of estrogen-dependent tumors [11]. However, studies have shown that for both *BRCA1* and *BRCA2* genes, the administration of these agents to prevent recurrence after disease onset led to a significant reduction in relapses. With regard to chemoprevention for ovarian cancer, numerous studies have demonstrated the effectiveness of combined oral contraceptive pills in preventing the onset of cancer in *BRCA* gene carriers [12].

In addition to *BRCA* carriers’ malignancy risk, prevention strategies and possible surgical and medical treatment needs, *BRCA* carriers face an underestimated concern of potent impaired fertility. *BRCA* carriage affects fertility both directly, due to the carriage itself, and indirectly, due to the malignancies and recommended preventive strategies associated with these genes. Thus, in the context of cancer prevention, performing a bilateral salpingo-oophorectomy, regardless of whether a mastectomy is performed, causes permanent loss of fertility if no fertility preservation procedure has been performed beforehand. In addition, in the event of malignancies associated with these genes, treatment has a significant impact on fertility. Furthermore, the effects of *BRCA* carriage on fertility are also evident in the psychological impact of carriage on women’s reproductive decisions. Specifically, the fear of transmitting the gene to offspring with the consequent impact of carriage on their health, the fear for the child’s future in the event of the woman herself developing cancer, as well as the fear of developing cancer due to pregnancy or during pregnancy, have a negative effect on women’s desire to have children. In addition, the pressure to find a suitable partner more quickly and complete family planning due to the recommendation for salpingo-oophorectomy is associated with further stress. Table 1 summarizes the main reproductive issues of clinical relevance in BRCA mutation carriers.

**Table 1:** Fertility issues in *BRCA* carriers.

Fertility Issues in *BRCA* Carriers
• Possible impairment of ovarian reserve • Increased likelihood of cancer during reproductive years • Gonadotoxicity of systemic cancer treatment • Recommendation for prophylactic salpingo-oophorectomy • Need for timely fertility preservation • Need for multidisciplinary counseling • Psychological issues

## 2. Materials and Methods

We conducted a narrative review incorporating studies of varying design and complexity. An electronic search of PubMed/MEDLINE and Scopus was performed between October 2025 and February 2026 to identify English-language publications examining the association between *BRCA* mutation carriage and infertility. The search strategy included combinations of the terms “*BRCA* carriers” OR “*BRCA* mutation carriers” AND “infertility” OR “fertility.” After removing duplicates, articles were screened for eligibility through evaluation of their abstracts and full texts. Additionally, reference lists of the selected studies were manually reviewed to identify further relevant publications that could contribute to the contextualization of our findings.

The search strategy, study selection, and data extraction were independently conducted by two researchers. Any disagreements regarding study inclusion were resolved through case-based discussion involving the two senior researchers.

## 3. Results

### 3.1. The Impact of BRCA Gene Carriage on Fertility

#### 3.1.1. Molecular and Histological Studies in *BRCA* Gene Carriers

Studies on the possible effect of *BRCA* carriers on a woman’s fertility began about two decades ago. However, research into the role of *BRCA* genes in breast cancer had begun much earlier. *BRCA* genes are known to be key genes involved in DNA repair, mainly through homologous recombination. Their function ensures the integrity of genetic material and the proper completion of the cell cycle. Incorrect DNA repair leads to genomic instability in all cells, including ovarian cells. During meiosis, genomic instability can lead to deleterious errors such as aneuploidy and even apoptosis [13]. A large study conducted in 2010 on *BRCA1* -mutated mice linked ovarian aging to this gene. Specifically, these mice had fewer and smaller follicles with significantly more double-stranded DNA fragments. They also reproduced less than normal mice [14]. Another study also conducted on mice in which the *BRCA1* gene had been deleted showed a reduced number of offsprings, decreased ovarian reserve, and impaired oocyte maturation [15]. With regard to the *BRCA2* gene, several studies have failed to demonstrate an association between the gene’s carriage and fertility disorders. This result may be related to the fact that the loss of the normal allele typically occurs later than that of the *BRCA1* gene, at an age when ovarian reserves are already declining, as well as the fact that the role of the *BRCA2* gene in DNA repair is comparatively smaller [13]. However, a study conducted on mice with a mutation in this gene demonstrated a high frequency of nuclear abnormalities and a significant reduction in oocytes. Nevertheless, these mice did not lose their ability to reproduce [16]. Finally, the *BRCA1* gene in particular has been found to be highly expressed in both male and female gametes as well as in embryos before implantation [17]. Dysfunction of these genes appears to affect embryogenesis and fertility. All those findings are yet to be studied in larger trials in humans, in order to prove the strength of this evidence.

In addition to its role in repairing mutations, the *BRCA1* gene has been implicated in telomere maintenance. Telomeres are repetitive DNA sequences located at the ends of chromosomes that protect genetic information during cell division. With each cell division, telomeres shorten until they are completely lost, at which point the cell eventually dies. Telomere shortening has also been associated with cellular aging due to accumulated exposure to oxidative stress. Telomere shortening is associated with genomic instability [18]. An experimental study conducted on mice in which telomere shortening had been achieved showed ovarian aging, which implied a possible connection between telomere shortening and ovarian impairment [19]. Another study conducted at the molecular level implied a correlation between the *BRCA1* gene and telomere integrity maintenance [20]. However, these results have not been demonstrated in studies of human ovarian tissue. Overall, the possible association between telomere maintenance and *BRCA1* gene along with the possible correlation between telomere shortening and ovarian aging suggests a potential but not yet confirmed role of *BRCA1* gene in premature aging of ovarian cells with a possible effect on ovarian reserves [18,20].

Furthermore, as part of research into ovarian aging in female carriers, a study was conducted in 2017 on ovarian tissue from female carriers who underwent prophylactic salpingo-oophorectomy and ovarian tissue from cadaveric material. The ovarian tissue was studied in terms of its density in primordial follicles and the percentage of double-strand DNA breaks. The results of this study show that *BRCA1* and *BRCA2* carriers had fewer primordial follicles per unit area, while DNA breaks were found to be higher only in *BRCA1* carriers. These findings were more pronounced in women over the age of 30. A limitation of this study was the small sample size of carriers, which was 18 [21]. Another study compared the number of oocytes per fragment and per surface area of ovarian tissue between *BRCA* carriers and non-carriers in women who underwent ovarian tissue cryopreservation before starting chemotherapy for breast cancer. The results of the study show that the number of oocytes was lower in carriers [22]. Table 2 summarizes the main findings of these two studies.

**Table 2:** Comparison of ovarian tissue between *BRCA* carriers and non-carriers.

Study	Sample Size	Main Findings	Notes	Limitations
*Lin et al., 2017* [21]	18 *BRCA* carriers 12 organ donor cadavers	Lower primordial follicle density in both *BRCA1* + 2 carriers Higher DNA DSBs in *BRCA1* carriers	Diminished ovarian reserve in *BRCA* carriers	Small sample size, only women >24 years old
*Lambertini et al., 2018* [22]	19 *BRCA* carriers 53 non-carriers	Lower number of oocytes per fragment and per mm^2^ in both *BRCA1* + *2* carriers	Reduced reproductive potential in *BRCA* carriers	Small sample size, no statistical significance was shown

The results of the above studies show that both *BRCA* genes, but especially the *BRCA1* gene, appear to affect ovarian reserves in terms of both the quantity and quality of oocytes. However, the studies mentioned above mainly concerned observations at the histological and molecular level. Other studies focused on observing the reproductive outcome in female carriers compared with women who do not carry any of the pathogenic mutations. The first large epidemiological study of patient-controls was published in 2010 and involved 2254 *BRCA* mutation carriers and 764 non-carrier controls, who were compared in terms of childbearing. The study took into account the possibility of infertility and the need for assisted reproduction. The results of this study show that there was no difference in the average number of births in the two groups, nor in the number of women who experienced reproductive difficulties [23]. Other published studies also showed no difference in fertility and parity between carriers and non-carriers [24,25,26]. A meta-analysis published in 2020, which included 13 studies, showed that *BRCA* carriers did not have higher rates of nulliparity [27]. Furthermore, a study of reproductive outcomes involving only *BRCA* gene carriers showed reassuring results in terms of spontaneous conceptions and live births. The women in this study were younger than the average age of childbearing in the country where the study was conducted, which sparked the hypothesis that especially at younger ages, *BRCA* carriage may not be associated with negative reproductive outcomes, which is why earlier childbearing is recommended [28]. However, another study showed fewer conceptions and full-term pregnancies in *BRCA1* carriers compared with women who were not carriers [29].

On the other hand, two large studies have indicated a reproductive advantage in *BRCA* carriers. The first study, published in 2011, showed that female carriers had an average of two more children than non-carriers. However, this study only included women born before 1975 [30]. The second study showed that female carriers were less likely to be childless, had a greater number of children, and had fewer miscarriages than non-carriers [31]. Regarding the risk of miscarriage in female carriers, data from another large study support the claim that female carriers are not at greater risk of miscarriage than non-carriers [32]. From the above, it is clear that despite the proven effects of *BRCA* gene mutations on ovarian cells, there is no clinical evidence that *BRCA* mutation carriers have fewer children than non-carriers. However, the above studies have limitations that prevent reliable conclusions from being drawn. In addition, many studies do not distinguish between *BRCA* carriers, as *BRCA1* and *BRCA2* carriers are included in the same group, which may affect the reliability of the results. Table 3 illustrates the possible mechanisms affecting reproductive function in BRCA carriers.

**Table 3:** Possible mechanisms affecting reproductive function in *BRCA* carriers.

Potential Mechanism	Possible Effect on Fertility
Impaired DNA repair	Genomic instability, DNA damage and apoptosis
Accelerated follicular depletion	Reduced ovarian reserve and ovarian aging
Impaired oocyte maturation	Reduced oocyte quality and meiotic abnormalities
Telomere dysfunction	Cellular aging and possible ovarian impairment
Impaired embryonic development	Possible effects on early embryogenesis

#### 3.1.2. Anti-Müllerian Hormone (AMH) Levels in *BRCA* Carriers vs. in Non-Carriers

Another factor associated with ovarian reserves that has been used as an indicator in studies on the effect of BRCA carriers on fertility is the serum AMH levels. AMH is a glycoprotein produced by the granulosa cells of the follicles in the early stages of their development, specifically in the preantral and antral stages. At a later stage of development, specifically when the follicle is selected as the dominant follicle, AMH production ceases. AMH produced by the follicles is released into the systemic circulation and maintains stable levels in the blood throughout the menstrual cycle. Its value is considered to reliably reflect the number of follicles and is used as a biomarker of ovarian reserve in women with fertility problems. It is also a reliable indicator of response to ovarian stimulation. The AMH levels in the blood circulation are influenced by various parameters, the most important of which is age, as it begins to decline naturally after the age of 25. Other factors that adversely affect its levels are smoking, obesity, and hormonal contraceptive methods. With regard to contraceptives, the decrease in AMH levels is temporary, and when they are discontinued, the AMH levels return to the age-appropriate normal range. In addition, AMH levels decrease following exposure to cytotoxic chemotherapy and may also be influenced by the presence of malignancy. Furthermore, methodological factors, including the assay platform used for AMH measurement, should be taken into consideration when interpreting and comparing AMH values across studies. Other conditions can increase AMH levels, such as polycystic ovary syndrome [33].

Based on the above data, many researchers compared the AMH levels of *BRCA* carriers with those of non-carriers in order to compare ovarian reserve. The results of these studies were contradictory. Several studies concluded that AMH levels of carriers were similar to those of non-carriers [33,34,35]. Most studies that observed a difference in AMH levels had differentiated *BRCA1* and *BRCA2* carriers into two different comparison groups, with *BRCA1* carriers more often characterized by lower AMH levels than *BRCA2* carriers and non-carriers [16,36,37] In these studies, no difference was found in AMH levels between *BRCA2* carriers and the control group. These findings are supported by a meta-analysis which confirmed lower serum AMH levels in *BRCA1* but not *BRCA2* mutation carriers, compared with non-carriers. Most of the women studied had been diagnosed with breast cancer [38]. The meta-analysis concluded that AMH levels in *BRCA1* carriers were approximately 23–25% lower compared with non-carriers.

Other studies have linked differences in AMH levels between carriers and non-carriers to age. Specifically, in two studies, the levels of serum AMH were found to be significantly lower in *BRCA1* carriers, but this reduction concerned specifically women over 35 years old. *BRCA2* carriers were not included in these studies [29,39]. However, two studies showed reduced AMH only in *BRCA2* carriers compared with *BRCA1* carriers and non-carriers [26,40]. One study compared AMH levels in *BRCA* carriers who underwent prophylactic salpingo-oophorectomy with their number of growing follicles. Surprisingly, no correlation was found, a result that led researchers to question whether AMH is ultimately the best indicator of ovarian reserve [15]. A large meta-analysis including data from ten studies demonstrated that women aged ≤42 years carrying *BRCA* mutations had significantly lower AMH levels compared with wild-type women. This association was particularly evident among *BRCA1* carriers, whereas no significant difference was observed between *BRCA2* carriers and non-carriers. These findings were observed in both healthy women and women affected by breast cancer [41]. From the above, it is clear that in studies conducted with AMH as a biomarker for comparison of ovarian reserve between carriers and non-carriers, the results are contradictory. However, in general most studies, including the meta-analyses mentioned, seem to agree that, especially in *BRCA1* carriers, the serum AMH levels are significantly lower than those of non-carriers in the same age group. In some studies, it is concluded that this decrease in AMH levels of carriers is only evident when comparing specific age groups of carriers and non-carriers. In *BRCA2* carriers, most studies show that AMH levels are comparable to those of non-carriers.

#### 3.1.3. Age of Menopause in *BRCA* Carriers vs. in Non-Carriers

Another parameter used as an indicator of earlier reduction in ovarian reserve was the age of menopause in carriers compared with non-carriers. A large study showed that in *BRCA1* and *BRCA2* carriers, the average age of menopause was statistically significantly lower than in non-carriers [42]. Another study also showed a younger age of menopause in carriers, but the difference appeared to be small and did not seem to affect fertility [25]. Finally, two large studies found no difference in the age of menopause between carriers and non-carriers [43,44]. However, considering that far fewer women ultimately reach natural menopause due to preventive salpingo-oophorectomy and cancer diagnosis at some stage of their reproductive life, it is more difficult to draw reliable conclusions. In fact, in the study mentioned above, only 19% of participants reached natural menopause, which affects the reliability of the results [43].

### 3.2. Assisted Reproduction in BRCA Carriers

Assisted reproduction techniques are often used in *BRCA* carriers. This particular population of women turns to these techniques to preserve their fertility before undergoing prophylactic salpingo-oophorectomy and before undergoing gonadotoxic treatment for breast cancer, which tends to occur earlier than in the general population. These techniques are also used due to the difficulty of natural conception after systemic cancer treatment, as well as for preimplantation diagnosis of *BRCA* carriage in offspring.

#### 3.2.1. Ovarian Stimulation in *BRCA* Carriers

The response to controlled ovarian stimulation is considered a reliable indicator of ovarian reserve and may help predict premature ovarian failure. One of the first studies to associate *BRCA* carriers with reduced ovarian reserve examined the response to ovarian stimulation in women with breast cancer, both *BRCA* carriers and non-carriers [45]. This study found that in *BRCA1* carriers, the number of oocytes collected was significantly lower than in women who were not carriers. No difference was observed in *BRCA2* carriers, but the sample size of these women was too small to draw reliable conclusions. Subsequent studies reached similar conclusions [22,46,47]. These studies found that in women with breast cancer who were *BRCA* carriers, the number of oocytes retrieved and the number of mature oocytes, i.e., the oocytes that had reached metaphase II and were subsequently cryopreserved, was lower than in patients who were not carriers. In the studies by Lambertini et al. and Porcu et al., carriers also required higher doses of gonadotropins during ovarian stimulation, and their AMH levels were significantly lower [22,46]. In the first study, these results concerned both *BRCA1* and *BRCA2* carriers, while in the second study they concerned only *BRCA1* carriers. In the study by Kim et al., *BRCA1* and *BRCA2* carriers were not studied separately [47]. Furthermore, in a study comparing women with the aforementioned characteristics, the results show that *BRCA1* and *BRCA2* carriers produced a corresponding number of oocytes compared with non-carriers, but the number of mature oocytes suitable for cryopreservation was lower [48]. In addition to women with breast cancer, *BRCA* carriers were also associated with a lower ovarian response in healthy women. A study concerning women who underwent in vitro fertilization for preimplantation diagnosis due to mutations and compared *BRCA* carriers with carriers of other mutations showed that *BRCA1* carriers produced fewer mature oocytes and also required higher doses of gonadotropins for follicular maturation. Furthermore, they ultimately achieved a lower number of pregnancies. *BRCA2* carriers did not show a lower ovarian response [49].

On the other hand, several studies suggested that the response to ovarian stimulation among *BRCA* mutation carriers was similar to that observed in non-carriers [34,35,50,51]. These studies included both women with breast cancer and healthy carriers who wanted preimplantation diagnosis or fertility preservation. They showed a similar number of oocytes retrieved and number of mature oocytes or embryos cryopreserved compared with non-carriers. In addition, some studies showed a similar number of achieved pregnancies [35,50]. Furthermore, two meta-analyses from 2020 showed a corresponding ovarian response between *BRCA* carriers and non-carriers. In the meta-analysis by Cordeiro et al., it appeared that carriers produced fewer but not statistically significantly fewer mature oocytes than non-carriers, while in the meta-analysis by Hu et al., the only difference between carriers and non-carriers was the smaller number of primordial follicles in carriers [50,52]. Furthermore, in a study comparing the rates of in vitro maturation and cryopreservation of oocytes between carriers and non-carriers, no difference was found between the populations compared. The women in this study underwent emergency immature oocyte retrieval for fertility preservation due to the need for immediate initiation of chemotherapy for breast cancer [53]. From the above, it can be concluded that ovarian stimulation in *BRCA* carriers is effective, although it is not clear whether it is as effective as ovarian stimulation in women who are not carriers. Further research with multicenter studies and meta-analyses with less heterogeneous characteristics than existing ones will provide clearer answers to this comparison.

As already mentioned, assisted reproduction techniques have been widely used to preserve fertility in women with cancer, especially breast cancer. Specifically, in modern society, with later childbearing and earlier age of onset of this disease in carriers compared with the general population, more and more cases of cancer are occurring in women who have not completed their family planning. In addition, at younger ages, and especially in *BRCA* carriers, there is an increased risk of developing malignancies with more aggressive biological characteristics that require cytotoxic chemotherapy with negative effects on fertility. An additional obstacle to reproduction is the recommendation to delay conception for approximately two years after completing treatment to reduce the risk of recurrence, which leads to a further reduction in ovarian reserves, which particularly affects women who are nearing the end of their reproductive life.

A question faced by both the scientific community and the patients themselves is whether it is safe for women with breast cancer to receive ovarian stimulation treatment, given that hormone therapy is generally contraindicated in this population. In the past, ovarian stimulation was not recommended for these women, except for cryopreservation of eggs obtained during a natural cycle. However, the number of eggs obtained in this way was very small and the chances of pregnancy were low. Thus, protocols involving the administration of letrozole and tamoxifen, which combine follicular maturation and anti-estrogenic action, together with gonadotropins, were introduced [54]. This protocol is considered safe and effective in women with breast cancer [55]. Although there is a delay in starting treatment, studies show that this does not appear to affect disease-free interval and survival [56]. The goal is to avoid a large increase in estrogen, which in classic protocols increases more than tenfold compared with the upper estrogen levels achieved in a natural cycle. There is no clear evidence that the temporary increase in estrogen caused by conventional ovarian stimulation protocols adversely affects oncological outcomes; however, controlled estrogen elevation is preferred in these patients. In particular, the combination of letrozole with follicle-stimulating hormone (FSH) is considered to achieve the lowest upper estrogen concentrations, similar to those of the natural cycle, with a greater number of oocytes and embryos, corresponding to those produced with conventional protocols, while there does not appear to be an increase in recurrence in these women compared with those who have not received fertility preservation treatment. This protocol is now widely used and known as COST-LESS [54]. In summary, women undergoing this treatment begin taking letrozole on the 2nd–3rd day of their cycle. After two days, FSH administration begins at a dosage proportional to age and ovarian reserves. To avoid premature luteinizing hormone (LH) surge and ovulation before the follicles are sufficiently mature, a gonadotropin releasing hormone (GnRH) antagonist is administered when the follicles reach 13 mm. When at least two follicles reach 20 mm, stimulation of final egg maturation begins with human chorionic gonadotropin (hCG), and after 34–36 h, the eggs are retrieved transvaginally. After retrieval, letrozole administration is continued until estrogen levels have fallen sufficiently. In a study, alternatively to hCG, the GnRH analogue leuprorelin was used to induce ovulation before subsequent suppression. The results of the study show that in patients who received leuprorelin compared with those who received hCG, estrogen levels decreased significantly more after ovulation, such that no subsequent continuation of letrozole was required, as in the classic protocol. In addition, there were fewer cases of ovarian hyperstimulation. Furthermore, a greater number of mature oocytes that had reached metaphase II and a greater number of embryos were produced [57].

Many studies have focused on evaluating the safety and efficacy of both ovarian stimulation in general and this specific protocol with letrozole in particular. A study of cancer patients who received an ovarian stimulation protocol with letrozole combined with recombinant FSH and were compared with patients who received a protocol with recombinant FSH alone showed that in women who received letrozole, the number of total and mature oocytes was higher, as was the number of embryos [58]. A meta-analysis comparing 990 women who received letrozole during ovarian stimulation with 1, 131 women who underwent ovarian stimulation without the addition of letrozole—and included both cancer patients and healthy patients—showed that the addition of letrozole is associated with comparable efficacy, that is, a comparable number of mature oocytes retrieved, with a significant reduction in peak estrogen levels [59]. A prospective study in women with breast cancer who underwent controlled ovarian stimulation with letrozole and were compared with patients who did not receive any treatment showed that the risk of recurrence and 5-year survival did not differ significantly between the two groups. Furthermore, this study showed no difference in recurrence and survival between the compared groups depending on *BRCA* status, malignancy characteristics (e.g., hormone sensitivity), and whether stimulation was performed before or after surgery [60]. In particular, there was no difference in disease-free survival or overall survival between *BRCA* carriers and non-carriers who underwent ovarian stimulation. Also, no difference was found when these parameters were compared between *BRCA* carriers who did and those who did not undergo ovarian stimulation.

Other studies compared *BRCA* carriers who underwent controlled ovarian stimulation due to infertility or desire for preimplantation diagnosis of *BRCA* carriage with *BRCA* carriers who did not undergo ovarian stimulation. In these studies, the patients were evaluated for the occurrence of breast cancer, and they also showed no increase in cancer risk in those who received fertility preservation treatment [61,62,63].

In addition to the cryopreservation of eggs or embryos just discussed, which is the first choice for fertility preservation in women with cancer who are about to undergo gonadotoxic chemotherapy, other options include ovarian tissue cryopreservation, in vitro oocyte maturation, and GnRH analogue administration before and during chemotherapy.

#### 3.2.2. Ovarian Tissue Cryopreservation in *BRCA* Carriers

Ovarian tissue cryopreservation is a good option in cases where the disease requires immediate therapeutic intervention and when ovarian stimulation is contraindicated. In addition to the advantage of time and avoiding ovarian stimulation treatment, it preserves the gonads intact and avoids the toxic effects of chemotherapy on them. It is also the only option for preserving fertility in prepubescent patients. However, two surgeries are required to perform this fertility preservation technique, one to remove the tissue and one to reimplant it after cryopreservation. In addition, in women with cancer, there is a risk of cancer cell dissemination during the reimplantation of ovarian tissue. In patients with breast cancer, there is little data on the application of this technique. A meta-analysis of the effectiveness of autologous cryopreserved ovarian tissue transplantation, which also included women with breast cancer, showed satisfactory effectiveness in terms of the number of pregnancies and live births [64]. For *BRCA* carriers, the data is even scarcer. Two successful pregnancies have been reported after reimplantation of cryopreserved ovarian tissue in two *BRCA2* carriers who developed breast cancer [22,65]. Due to ethical dilemmas concerning the incidence of ovarian cancer and lack of data, this technique is not widely recommended for carriers at present. It could be an option for young female carriers who are still many years away from the recommended age for salpingo-oophorectomy. However, even in this setting, removal of the transplanted ovarian tissue is recommended once childbearing has been completed. Another approach to ovarian tissue transplantation is to perform it heterotopically, such as in the forearm or the abdominal wall. The advantage of this method, especially for *BRCA* carriers, is that it allows for closer and easier monitoring of the tissue compared with its anatomical location. Furthermore, a study has shown that follicle development is possible even in an ectopic location and that oocyte retrieval can be performed [66]. Further research is needed to prove the effectiveness of this method. To date, the precise risk of developing cancer after ovarian tissue autotransplantation in *BRCA* mutation carriers remains unknown.

#### 3.2.3. In Vitro Maturation of Oocytes in *BRCA* Carriers

Another technique that could be applied in cases where immediate treatment for breast cancer is necessary or when hormone therapy is contraindicated is in vitro maturation of oocytes (IVM). This technique can be performed immediately and does not involve the exogenous administration of ovarian stimulation therapy. It is also an ideal choice for women at increased risk of ovarian hyperstimulation syndrome (OHSS). Another advantage of this method is that whether collection takes place in the follicular or luteal phase, the number of oocytes retrieved, matured, and cryopreserved appears to be similar, allowing for cycle-independent oocyte retrieval when it needs to be performed very quickly [67]. The oocytes that are retrieved by transvaginal aspiration are immature, and mature in vitro to metaphase II, where they are then cryopreserved. However, the number of cells that will ultimately mature is unpredictable, and studies have shown that in vitro maturation of oocytes is less effective than ovarian stimulation in terms of the number of oocytes cryopreserved [68,69]. There is limited data concerning the efficacy of this method in *BRCA* carriers. According to a study comparing the IVM outcomes between *BRCA* carriers and non-carriers, the results were similar. In this study, the number of oocytes retrieved and reaching metaphase II in *BRCA* carriers was comparable to that in non-carriers [53]. In addition, the possibility of in vitro maturation of oocytes obtained from ovarian tissue removed prior to chemotherapy has been investigated with encouraging results [70]. For greater effectiveness, cryopreservation of ovarian tissue is often combined with this technique [69]. Especially for *BRCA* carriers, the possibility of maturing and cryopreserving oocytes without replacing the ovarian tissue offers significant advantages. Table 4 summarizes the main indications, advantages and disadvantages of each fertility preservation option.

**Table 4:** Comparison of fertility preservation options.

Fertility Preservation Options	Indications	Advantages	Disadvantages
Oocyte/embryo cryopreservation	First line option in fertility preservation	Higher efficacy compared with other methods, considered safe	Requires time for ovarian stimulation, risk of OHSS, may be contraindicated (i.e., in patients with increased thromboembolic risk)
Ovarian tissue cryopreservation and reimplantation	Young, prepubertal patients, lack of time, contraindication of ovarian stimulation	Does not require time, suitable when ovarian stimulation is contraindicated, intact gonads after chemotherapy	Unknown risk of ovarian cancer, risk of reintroduction of cancer cells, two surgeries required, recommendation to remove ovarian tissue after completion of family planning
In vitro maturation of oocytes	Lack of time, high risk of OHSS, contraindication of ovarian stimulation	Does not require time, no surgery, no ovarian simulation, no need for menstrual cycle synchronization	Lower efficacy, has been linked to increased risk of aneuploidies
GnRH analogues	Premenopausal women undergoing gonadotoxic chemotherapy	Gonadal protection, reduces risk of premature ovarian insufficiency	Should not be considered a substitute for established fertility preservation methods and should preferably be combined with oocyte or embryo cryopreservation whenever feasible

### 3.3. Ovarian Toxicity Induced by Chemotherapy

As already mentioned, *BRCA* carriers often develop malignancies with more aggressive biological characteristics that require chemotherapy. Especially in young women, such treatment can be devastating for the gonads, as it can lead to complete ovarian failure, resulting in amenorrhea, menopausal symptoms, and osteoporosis. Laboratory tests show increased gonadotropins and decreased estrogens. Histologically, ovarian atrophy, stromal fibrosis, and absence of primordial follicles are observed. In cases where ovarian failure is temporary due to partial damage to the ovaries, the menstrual cycle usually returns within six months. As chemotherapy targets dividing cells, it destroys follicles in the maturation phase, causing apoptosis in granulosa cells. Morphological and genetic abnormalities are caused in the oocytes, while their response to ovarian stimulation is reduced [71]. In primordial follicles, the effect depends on the chemotherapeutic agent [72]. If pregnancy is achieved during chemotherapy, the outcome depends on the stage of pregnancy. In the first four weeks, if the embryo is affected, it is often aborted. Up to ten weeks, damage to the embryo can cause teratogenesis, while later it can lead to neurological abnormalities, IUGR, prematurity, and stillbirth [73,74] Pregnancy after completion of chemotherapy, particularly after an interval of approximately one year, has not been associated with an increased risk of miscarriage or congenital abnormalities according to the available evidence [75]. These data concern women with cancer in general and are not specific to *BRCA* mutation carriers.

The degree of toxicity to the ovaries depends on age, existing ovarian reserve, the chemotherapeutic agent, and its dosage [76]. Anthracyclines, taxanes, and especially alkylating agents, which are key chemotherapy options in breast cancer, are considered gonadotoxic. With regard to *BRCA* carriers, there are limited and conflicting data regarding the impact of chemotherapy on ovarian reserve compared with non-carriers. In a 2013 study by Valentini et al., chemotherapy-induced amenorrhea was compared as a clinical indicator of gonadotoxicity between carriers and non-carriers, and no difference was found between the subgroups [77] In the 2019 study by Lambertini et al., *BRCA* carriers and non-carriers were compared based on their AMH levels according to the chemotherapy regimen and the addition of tamoxifen [71] In all subgroups, *BRCA* status did not appear to affect patients’ AMH levels. In contrast, other evidence suggests a greater decline in ovarian reserve among *BRCA* mutation carriers following chemotherapy compared with non-carriers [78]. These discrepancies are likely related to heterogeneity in study design, chemotherapy agents and protocols, and the markers used for gonadal function evaluation (e.g., AMH vs. chemotherapy-induced amenorrhea). Further studies are needed to establish firm conclusions.

The addition of GnRH analogues to suppress the ovaries during chemotherapy in women with breast cancer is considered protective whether the cancer is hormone-sensitive or not. Large randomized studies have demonstrated significant protection against premature ovarian failure as well as a higher number of pregnancies in women who received this adjunctive therapy [79,80]. However, due to insufficient data on the outcome of subsequent pregnancies, while their use in combination with chemotherapy is recommended for all premenopausal women, replacing cryopreservation with this technique for fertility preservation is not recommended. For *BRCA* carriers, there is insufficient data on the protection of GnRH analogues during chemotherapy against premature ovarian failure, but it is recommended that they be taken in the same way as in non-carriers [81]. Administration should begin at least one week before the start of chemotherapy and continue throughout its duration.

With regard to targeted anti-Human Epidermal Growth Factor Receptor 2 (anti-HER2) therapy and newer therapies such as poly ADP ribose polymerase (PARP) inhibitors and immunotherapy, there is insufficient data on their effect on fertility. Similarly, there is little data on the effect of hormone therapy on ovarian reserves, but it does not appear to have a significant effect on its own [82,83]. Some studies have shown that adding tamoxifen to chemotherapy increases the toxic effect on the ovaries, but this has not been confirmed by other studies [75,77]. Hormone therapy is contraindicated during pregnancy. Since hormone therapy is recommended for 5–10 years, fertility is significantly affected due to a decrease in ovarian reserves during this period. However, studies investigating the oncological outcomes in women who discontinue hormone therapy for a period of approximately two years to achieve pregnancy show encouraging results [84].

In any case, immediate referral to a reproductive specialist after a cancer diagnosis is of utmost importance. The time between therapeutic interventions (surgical planning, initiation of chemotherapy) is usually sufficient to perform the appropriate fertility preservation method, even ovarian stimulation with egg retrieval. The earlier the referral, the more cycles of ovarian stimulation can be performed with more favorable results in preserving fertility.

### 3.4. Preimplantation Diagnosis of BRCA Carriage

Finally, *BRCA* carriers, as mentioned, turn to assisted reproduction methods because of their desire to control the transmission of the *BRCA* gene to their offspring. Preimplantation genetic testing for monogenic disorders (PGT-M) is an approved indication for *BRCA* mutation carriers according to the European Society of Human Reproduction and Embryology Ethics Taskforce [85]. This method involves embryo biopsy, most commonly through trophectoderm biopsy at the blastocyst stage, followed by molecular genetic analysis to identify embryos carrying the pathogenic variant before implantation [86]. The results regarding the health of children born using this method are encouraging [87]. However, there is psychological and ethical division regarding preimplantation diagnosis for these specific genes, both among the scientific community and among the parents themselves. This is because these specific genes are not lethal, do not affect human functionality, and are not 100% related to the onset of cancer, creating ethical and emotional dilemmas in the decision to reject the embryo [88]. Another way to control the transmission of the mutated gene is prenatal diagnosis by taking chorionic villi or amniocentesis, which can be done relatively early in pregnancy (end of the first trimester to the beginning of the second). As mentioned above, the emotional and ethical dilemmas involved, especially at this stage of pregnancy, do not usually lead to termination of the pregnancy in cases of carrier status.

## 4. Conclusions

In summary, as far as the impact of *BRCA* gene carriage on fertility is concerned, studies conducted at the molecular and histological levels have shown the negative effect of *BRCA* mutations, especially *BRCA1* , on ovarian reserve. However, this impact is not reflected in the reproductive outcomes of carriers compared with non-carriers. Regarding anti-Müllerian hormone, which is a reliable marker of ovarian reserve, most studies demonstrate a decrease in its levels, particularly in *BRCA1* carriers; however, this is not a consistent finding. Finally, studies on the age of menopause in carriers also show mixed results; however, this indicator is less reliable because many women do not ultimately reach natural menopause. The inconsistent findings across studies are likely due to population heterogeneity, unaccounted confounding factors, small sample sizes, and differences in the BRCA mutations investigated. Further large-scale studies are needed to clarify these associations.

Assisted reproduction techniques are appropriate for *BRCA* carriers, as these women often need to preserve their fertility. Studies comparing the ovarian response of carriers after stimulation with that of non-carriers have yielded conflicting results. Specifically, while several studies have shown a reduced response to ovarian stimulation in *BRCA* carriers, particularly *BRCA1* carriers, other studies, including meta-analyses, do not confirm this finding and argue that ovarian stimulation is equally effective in carriers and women who do not carry pathological mutations. Ovarian stimulation and cryopreservation of oocytes or embryos in *BRCA* carriers, whether due to cancer or infertility, does not appear to increase the risk of developing or recurring breast cancer and is indicated when there’s enough time and no further contraindications.

Other fertility preservation techniques (cryopreservation of ovarian tissue, in vitro oocyte maturation, and addition of GnRH analogues to chemotherapy) are indicated as in non-carriers, with a recommendation to avoid reimplantation of cryopreserved ovarian tissue, especially in women who are close to the recommended age for salpingo-oophorectomy. The few studies that have been conducted do not indicate that chemotherapy-induced gonadotoxicity has a greater effect on *BRCA* carriers.

Finally, *BRCA* carriers now have the option of resorting to assisted reproduction methods for preimplantation diagnosis of the carrier status in their offspring, thereby avoiding transmission of the pathological gene.

## 5. Future Directions

In conclusion, *BRCA* mutation carriers face several reproductive challenges. The trend toward delayed childbearing in modern society, the higher incidence of malignancies at a younger age, and the recommended risk-reducing surgeries highlight the importance of timely fertility counseling. Furthermore, evidence suggesting a detrimental effect of *BRCA* mutations, particularly *BRCA1* , on ovarian reserve, together with the lower AMH levels reported in many studies, further support early discussion of fertility preservation options. Ultimately, reproductive counseling and decision-making should be individualized, taking into account the woman’s age, *BRCA* mutation status, cancer status, planned risk-reducing surgery, reproductive goals, and personal preferences.

**Key points**:

1. Current evidence suggests that BRCA1 pathogenic variants may be asso-ciated with impaired DNA repair mechanisms, accelerated ovarian ageing, and reduced ovarian reserve.
2. BRCA carriers do not appear to have reduced reproductive performance or higher rates of infertility compared with non-carriers.
3. Most studies report lower AMH levels in BRCA carriers, particularly BRCA1 car-riers, compared with non-carriers.
4. Age at menopause is not a reliable indicator of premature ovarian ageing in BRCA carriers.
5. BRCA2 carrier status does not appear to significantly affect the above parameters.
6. Where not contraindicated, oocyte and embryo cryopreservation is the first-line fertility preservation option in healthy carriers or patients with breast cancer, and does not appear to increase the risk of breast cancer occurrence or recurrence.
7. Ovarian tissue cryopreservation and transplantation is not a widely accepted fer-tility preservation technique in BRCA carriers due to potential oncological risks; however, it may be applied under specific conditions.
8. Oocyte in vitro maturation (IVM) is a less effective fertility preservation tech-nique, which may be used when hormonal stimulation is contraindicated, at any phase of the menstrual cycle.
9. GnRH analogues may be offered to women undergoing chemotherapy to reduce the risk of treatment-induced ovarian insufficiency; however, they should not re-place established fertility preservation techniques.
10. Cytotoxic chemotherapy appears to affect BRCA carriers in a similar manner compared with non-carriers.
11. Preimplantation genetic testing for BRCA pathogenic variants may be performed to prevent transmission of the mutation to offspring.

## Data Availability

No new data were created or analyzed in this study. Data sharing is not applicable to this article.
